# Rules of parotid gland dose variations and shift during intensity modulated radiation therapy for nasopharyngeal carcinoma

**DOI:** 10.1186/s13014-014-0307-2

**Published:** 2015-01-08

**Authors:** Wei Wang, Haihua Yang, Yucheng Mi, Wei Hu, Weijun Ding, Youyou Xie, Yujie Cai, Xiaofeng Chen

**Affiliations:** Department of Radiation Oncology, Affiliated Taizhou Hospital of Wenzhou Medical University, Zhejiang, 317000 China; Laboratory of Cellular and Molecular Radiation Oncology, Affiliated Taizhou Hospital of Wenzhou Medical University, Zhejiang, 317000 China; Department of Radiology, Affiliated Taizhou Hospital of Wenzhou Medical University, Zhejiang, 317000 China; Enze Medical Research Center, Affiliated Taizhou Hospital of Wenzhou Medical University, Zhejiang, 317000 China

**Keywords:** Nasopharyngeal carcinoma, Intensity-modulated radiotherapy, Parotid gland

## Abstract

**Background:**

To determine the position and dose delivery changes rules of parotid gland (PG) during the course of intensity modulated radiation therapy (IMRT) in nasopharyngeal carcinoma patients (NPC).

**Materials and methods:**

One hundred and forty one competed tomography (CT) images from 47 NPC patients (three images for each patient were acquired before treatment, at the 15th and 25th fraction during the treatment) who underwent radical IMRT were selected for this study. A total of 70-76Gy at 2.12–2.3 Gy/fraction/d was given to the GTVnx in 33 fractions. The distances between the lateral/medial aspects of PG and midline (DLM and DMM) at the level of odontoid process were measured. The dose differences between plan and actual delivery were calculated.

**Results:**

The volume reductions of PGs between fractions 15 and 25 were larger than those in the first 15 fractions (4.68 ± 3.23 cc vs. 2.46 ± 4.55 cc for the right PG and 5.96 ± 2.99 cc vs. 2.06 ± 2.99 cc for the left PG). However, the percentage of gland volume receiving ≥30 Gy (V30) of bilateral PGs decreased more significantly in the first 15 fractions than that between fractions 15 and 25 (5.61 ± 16.04% vs. 1.14 ± 21.54% for the right PG and 6.87 ± 15.58% vs. 0.81 ± 15.94% for the left PG). The gross tumor volume of the nasopharynx (GTVnx) decreased more significantly in the first 15 fractions than that between the 15th and 25th fraction (8.23 ± 13.61 cc vs. 3.30 ± 8.09 cc). The DMM of ipsilateral PGs reduced in the first 15 fractions (0.80 ± 2.96 mm) but increased between fraction 15 and 25 (−2.19 ± 3.96 mm). While ipsilateral PG shifted into target volume but shifted out target volume between fraction 15 and 25. Parotid glands V30 was correlated with GTVnx, GTVnx reduction and DMM reduction (p < 0.01).

**Conclusion:**

Our results indicate that the reduction of GTVnx leads to the positional change of the parotid gland, which results in more significant dose change of the parotid gland in the first 15 fractions than that between fraction 15 and 25.

## Background

Nasopharyngeal carcinoma (NPC) is common in southern China [1]. Radiation therapy concurrently with chemotherapy is the definitive treatment for NPC [2,3]. Over the past decade, intensity-modulated radiotherapy (IMRT) has been widely delivered in clinic. Some patients receiving radiation therapy (RT) to the head and neck have significant anatomic changes during IMRT course, including shrinking nodal masses or primary tumors, resolving postoperative changes/edema, and changes in overall body habitus/weight loss [4-9]. It has been reported that replanning by using the second CT scan with an average interval of 19 fractions during the course of IMRT for head and neck cancer patients significantly reduced the normal organ dose and increased the target dose coverage compared with using the original plan on the new anatomy [8]. Our previous studies implicated that replanning was needed before the 25th fraction in 50% of IMRT plans to avoid the overdose to the normal sensitive structures [10,11]. We have recently reported that anatomic changes resulted in more predominant dosimetric effects in the first 15 fractions than those between fraction 16 and 25 [12].

Traditionally, IMRT plan was generated from single 3D anatomy prior to treatment. For the head and neck tumor, one of the most important roles of IMRT is to spare the function of parotid gland, which may potentially alleviate the degree and the rate of xerostomia [13,14]. Several studies have reported that bilateral parotid glands had a significantly variance of volume reduction over the treatment [11,12]. A recent study [15] investigated 10 patients with locally advanced oropharyngeal cancer treated with contralateral parotid-sparing IMRT concurrently with platinum-based chemotherapy. And it has been found that ipsilateral and contralateral parotids showed a mean reduction in volume of 29.7% and 28.4%, respectively. Those volume reductions resulted in significant dose changes. To date, there are very limited studies on parotid gland position and dose changes in IMRT of nasopharyngeal carcinoma. Therefore, we carried out this study to evaluate the rules of dose delivery changes and shift of parotid gland during IMRT for NPC.

## Methods and materials

### Patients

From November 2008 and December 2011, forty-seven NPC patients who underwent radical IMRT were selected for this study. This study was approved by the Institutional Review Board, and informed consent was obtained from all patients before entering the study. Patient and tumor characteristics are shown in Table 1.

**Table 1:** **Baseline characteristics of the study patients**

		**N**
**Age (yrs)**	**Medial: 51 (range 28 ~ 71)**	
Gender	Male:	29
	Female:	18
T	1	13
	2	19
	3	7
	4	8
N	0	8
	1	16
	2	18
	3	5
Stage	I	4
	II	15
	III	17
	IVa	6
	IVb	5

### Prospective image and treatment planning

Patients were treated in the supine position and immobilized with a custom thermoplastic mask covering the head, neck and shoulders. Simulation CT scans for the initial treatment plans (SCT-1) were performed with contrast medium at 2.5 mm slice intervals from the skull vertex to 2 cm below the clavicles. Two re-simulation CT scans were performed at the 15th (SCT-2) and 25th fraction (SCT-3) of IMRT for replanning. A total of 141 CT simulation scans were obtained over the study period.

The gross tumor volumes (GTVs) included the primary nasopharyngeal tumor (GTVnx) and involved lymph nodes (GTVnd), as shown by clinical information and endoscopic and radiologic examinations (including CT and MRI). The clinical target volume (CTV) included the high-risk regions (CTV1) and the low-risk regions (CTV2). Detailed target definition and dose constraints for IMRT planning have been described in our prior study [10]. A total of 70–76 Gy (2.12–2.3 Gy/fraction), 66–70 Gy (2.0–2.12 Gy/fraction), 60–66 Gy (1.8–2.0 Gy/fraction), and 56–60 Gy (1.7–1.8 Gy/fraction) were delivered to PTVs of the GTVnx, GTVnd, CTV1, and CTV2, respectively, in 33 fractions with simultaneous integrated boost. All IMRT plans were designed by inverse planning with commercial treatment planning systems (Corvus 6.2 version, NOMOS Corporation).

Plan 1 was the initial IMRT plan generated based on the SCT-1. Plan 3 was the re-IMRT plan on the SCT-2. Plan 2 was made by applying the beam configurations of plan 1 to SCT-2 and Plan 4 was made by applying the beam configurations of plan 3 to SCT-3 for each patient. For each IMRT Plan, Dose-volume histograms (DVHs) were calculated for parotid glands. The percentage of gland volume receiving ≥ 30 Gy (V30) of PGs were evaluated in the four plans.

### Measurement of parotid gland volume

Organs at risk (OARS) such as parotid glands were manually delineated on each axial slice of the pre-treatment planning CT. CT-CT fusion was used to decrease the inter-fractions step-error. Each parotid gland was outlined on the fusion slides as described previously [16] by the same physician/radiologist. Volumes of bilateral PGs were compared between SCT-1 and SCT-2, SCT-2 and SCT-3 for each patient.

### Measurement of parotid gland shift and transverse diameter

**Figure 1:**
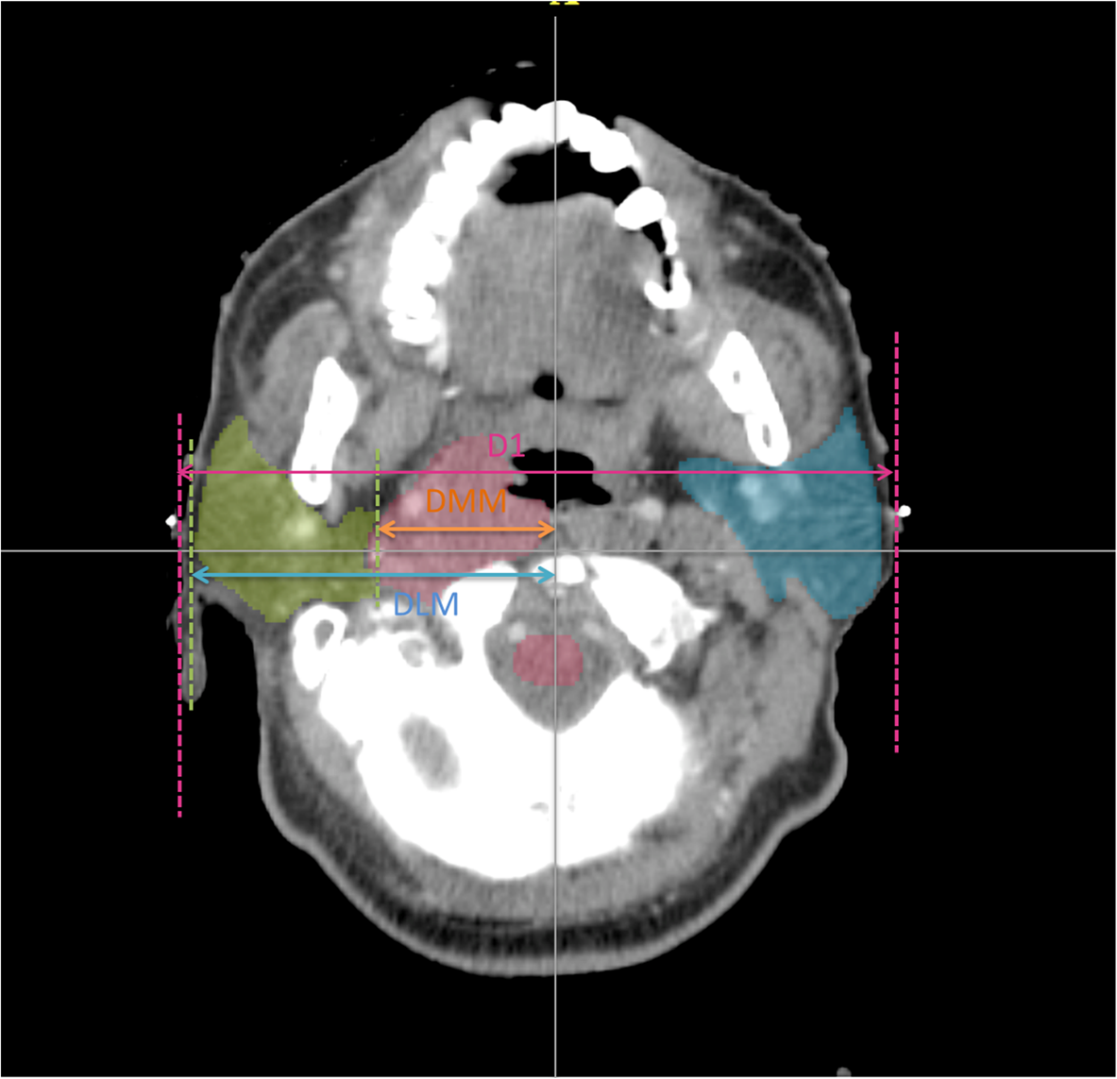
**Definition of measurement of parotid gland shift and transverse diameter. DLM** represents the distance between the lateral aspects of the parotid gland and the midline on the most superior slice with the odontoid process just still visible. **DMM** represents the distance between the medial edge of the parotid gland to the midline on the most superior slice with the odontoid process just still visible. The transverse diameter of the nasopharyngeal level **(D1)** represents the distance between the intersection points on both sides of skin edges, at the level of the odontoid process. It is a posterior marginal connection for the bilateral mandibular angle [10].

To quantify the positional shifts of the parotid glands, the distances between the lateral/medial sides of PG and midline (DLM and DMM) at the level of odontoid process were measured by using the methods we have previously described [10] (Figure 1).

### Data statistical analysis

The Statistical analysis was performed using software package SPSS (version 13.0 software; SPSS Inc., Chicago, IL). A paired- samples *t*-test was used to compare volumes and shift of bilateral parotid glands between SCT-1 and SCT-2, SCT-2 and SCT-3, and doses of bilateral parotid glands between Plan 1 and Plan 2, Plan 3 and Plan 4. Associations between continuous variables were analyzed by Pearson’s correlation test. A p <0.05 was considered statistically significant.

## Results

### Patients and image information

The characteristics of the patients are shown in Table 1. A total of 141 SCTs from 47 nasopharyngeal carcinoma patients were included this study.

### Changes of target and parotid gland volume

The average volume of GTVnx was 27.37 ± 28.79 cc, 18.49 ± 21.48 cc and 16.36 ± 17.70 cc on SCT-1, SCT-2 and SCT-3. And the average volume of GTVnd was 27.01 ± 38.06 cc, 17.75 ± 28.93 cc and 13.35 ± 23.34 cc on SCT-1, SCT-2 and SCT-3. As shown in Table 2, the GTVnx and GTVnd volumes had more significant reduction on the SCT-2 compared with SCT-3. The average parotid gland volume was 21.34 ± 6.50 cc, 19.08 ± 5.50 cc, and 13.79 ± 5.15 cc on SCT-1, SCT-2 and SCT-3. Unlike the changes of GTVnx and GTVnd volumes, the mean volume changes in parotid glands had more significant decrease on the SCT- 3 compared with on SCT-2. Similar change trends were found in comparing with the ipsilateral parotid gland (IP-OG) and the contralateral parotid gland (CON-PG).

**Table 2:** **Volume and dose changes of target and parotid gland**

	**The first 15 fractions**			**Between fraction 15 and 25**		
	**SCT-1 vs. SCT-2**			**SCT-2 vs. SCT-3**		
**Volume changes**	**Mean ± SD**	**95% CI**	***p*** **Value**	**Mean ± SD**	**95% CI**	***p*** **Value**
GTVnx	8.23 ± 13.61	4.24; 12.24	0.000	3.30 ± 8.09	0.92; 5.67	0.008
GTVnd	10.27 ± 13.37	6.02; 14.51	0.000	4.88 ± 8.41	2.19; 7.57	0.001
L-PG	2.06 ± 2.99	1.19; 2.94	0.000	5.90 ± 2.99	5.02; 6.78	0.000
R-PG	2.46 ± 4.50	1.14; 3.78	0.000	4.68 ± 3.23	3.74; 5.63	0.000
CON-PG	2.98 ± 3.50	1.80; 4.17	0.000	5.78 ± 3.00	4.77; 6.80	0.000
IP-PG	1.81 ± 3.94	0.78; 2.85	0.001	4.99 ± 3.23	4.14; 5.84	0.000
**Dose changes**	**Plan 1 vs. Plan 2**			**Plan 3 vs. Plan 4**		
**(PG V30)**	**Mean ± SD**	**95% CI**	***p*** ** Value**	**Mean ± SD**	**95% CI**	***p*** **Value**
L-PG	−6.87 ± 15.58	−11.45; −2.29	0.004	−0.81 ± 15.94	−5.49; 3.87	0.728
R-PG	−5.61 ± 16.04	−10.32; −0.90	0.021	−1.14 ± 21.54	−7.46; 5.19	0.719
CON-PG	−3.41 ± 13.44	−8.18; 1.36	0.155	0.25 ± 21.61	−7.42; 7.91	0.948
IP-PG	−7.77 ± 16.76	−12.06; −3.48	0.001	−1.64 ± 17.33	−6.07; 2.80	0.464

*Abbreviations*: *SD* standard deviation, *CI* confindence interval, *R-PG* the right parotid gland, *L-PG* the left parotid gland, *GTVnx* the gross tumor volume of the nasopharynx, *GTVnd* the cervical lymph node, *Con-PG* contralateral parotid gland, *IP-PG* ipsilateral parotid gland, *V30* the percentage of parotid gland volume receiving ≥ 30Gy, *SCT-1* the first simulation CT scan before treatment, *SCT-2* the second simulation CT scan at the fifteenth fraction of treatment, *SCT-3* the third simulation CT scan at the twenty-fifth fraction of treatment, *Plan 1* the initial IMRT plan generated based on the SCT-1, *Plan 2* was made by applying the beam configurations of Plan 1 to SCT-2, *Plan 3* the re-IMRT plan on the SCT-2, Plan 4 was made by applying the beam configurations of Plan 3 to SCT-3.

### Variation of parotid glands V30

The mean changes in parotid glands V30 had more significant decrease on the plan 1 vs. plan 2 compared with on plan 3 vs. plan 4. The IP-PG was more remarkable in comparing with the CON-PG (Table 2).

### Displacement of parotid gland during IMRT

Table 3 shows lateral edge and medial edge of parotid gland shifts between SCT-1 and SCT-2, SCT-2 and SCT-3. The lateral edge of parotid glands had shifted more significantly on SCT-3 than on SCT-2. The lateral edge of contralateral parotid glands shifted with an average 0.47 ± 1.47 mm (p = 0.016) on SCT-2 compared with an average of 1.98 ± 1.83 mm (p = 0.000) on SCT-3. The lateral edge of ipsilateral parotid glands shifted with an average of 3.38 ± 1.53 mm (p = 0.000) on SCT-2 compared with 1.65 ± 2.04 mm (p = 0.000) on SCT-3. The medial edge of contralateral parotid gland shifted with an average of −0.53 ± 2.80 mm (p = 0.000) on SCT-2 compared with an average of −2.49 ± 3.22 mm (p = 0.000) on SCT-3. But, the medial edge of ipsilateral parotid gland shifted with an average of 0.80 ± 2.96 mm (p = 0.038) on SCT-2 compared with an average −2.19 ± 3.96 mm shift with on SCT-3. Thus the ipsilateral parotid gland moved to the target volume on SCT-2.

**Table 3:** **Position changes of parotid gland during intensity modulated radiotherapy**

	**The first 15 fractions**			**Between fraction 15 and 25**		
	**SCT1 vs. SCT2**			**SCT2 vs. SCT3**		
	**Mean ± SD**	**95%CI**	***p*** **Value**	**Mean ± SD**	**95%CI**	***p*** **Value**
Positional changes (mm)						
All-PG						
DLM	2.65 ± 2.12	2.21; 3.09	0.000	1.77 ± 1.96	1.37; 2.11	0.000
DMM	−1.25 ± 4.21	−2.12; −0.39	0.005	−0.56 ± 3.29	−1.24; 0.11	0.100
Con-PG						
DLM	0.47 ± 1.07	0.09; 0.85	0.016	1.98 ± 1.83	1.33; 2.63	0.000
DMM	−0.53 ± 2.80	−1.52; 0.46	0.281	−2.49 ± 3.22	−3.63; −1.35	0.000
IP-PG						
DLM	3.38 ± 1.53	3.44; 4.23	0.000	1.65 ± 2.04	1.13; 2.17	0.000
DMM	0.80 ± 2.96	0.047; 1.56	0.038	−2.19 ± 3.96	−3.21; −1.18	0.000

*Abbreviations*: *DLM* the distance between the lateral aspects of PG and midline, *DMM* the distance between the medial aspects of PG and midline, The other abbreviations are the same as those in the Table 2.

The positive and negative value for position changes means shift inside and outside, respectively.

### Correlation analysis of parotid glands V30

As shown in Table 4, V30 of parotid glands are significantly correlated with GTVnx volume (P = 0.002), GTVnx volume reduction (P = 0.006) and DMM reduction (P = 0.000). However, V30 of parotid glands are not correlated with GTVnd volume (P = 0.129), GTVnd volume reduction (P = 0.610) and DLM reduction (P = 0.118).

**Table 4:** **Correlation analysis of parotid glands V30 and volumetric and positional variations**

	**Pearson correlation**	**Sig. (2-tailed)**
GTVnx	0.226	0.002
GTVnx reduction	−0.200	0.006
GTVnd	−0.120	0.129
GTVnd reduction	0.041	0.610
PG volume	0.006	0.938
PG volume reduction	0.052	0.447
D1	0.052	0.482
D1 reduction	−0.097	0.184
DLM	0.089	0.224
DLM shift	−0.114	0.118
DMM	0.090	0.220
DMM shift	0.281	0.000

*Abbreviations*: *D1* represents the transverse diameter of the nasopharyngeal level. The other abbreviations are the sa me as the Table 2.

## Discussion

IMRT has been used routinely in the treatment of NPC due to its anatomical position. The sharp dose gradient change around the target margin in IMRT requires accurate dose delivery to the target volume and critical organs. However, it has been demonstrated that most NPC patients will experience anatomic changes, mainly including shrinking of the primary tumor or nodal masses, parotid glands during IMRT [8,9,11]. Furthermore, anatomic changes could have a potential dosimetric impact when a highly conformal IMRT technique is used.

We have previously demonstrated that replanning during IMRT for NPC significantly improved 2-year local regional control rate. Replanning had a profound impact on the quality of life of NPC patients. Patients who did not receive replanning had significantly greater problems with speech, social contact, teeth, mouth opening, dry mouth and sticky saliva [17]. Furthermore, it has been reported that IMRT replanning improved the 3 years local progression–free survival for NPC patients with AJCC staged of late T or N, whereas patients at early stage could not benefit from IMRT replanning by retrospectively analyzing 33 patients [18]. Since protection of parotid glands function directly affects the quality of life, dose variation rule of parotid gland becomes the focus of research in the field of IMRT for nasopharyngeal carcinoma.

The changes of volume and dose of parotid gland by repeating simulation of KV-CT scans through the IMRT for nasopharyngeal carcinoma have been analyzed in our previously studies. A significant larger reduction of parotid volume was found between fraction 16 and 25 than in the first 15 fractions. However, the dose changes of the parotid gland were more prominent in the first 15 fractions than those between fractions 16 and 25 during IMRT [12]. Similarly, in the current study, we found that the variation of parotid glands V30 was more significantly decreased during the first 15 fractions than between fraction 16 and 25, particularly for the ipsilateral parotid glands. Several studies have suggested that the dose change of parotid gland may result from the parotid gland volume change [9]. However, Some other studies suggested that the dosimetric changes may be related with the positional shifts of the parotid glands during IMRT treatment [4,6]. Nishi T et al. reported that parotid glands and the retromandibular vein in parotid glands shifted medially an average of 4.2 mm and 2.4 mm separately at the third or fourth week of IMRT for NPC [4]. In our present study, we further demonstrated that the changes of parotid glands V30 variation may be associated with more significant shrinkage for GTVnx and the parotid glands moving into the high-dose region at the first 15 fractions.

## Conclusion

In conclusion, our current study indicated that shrinking of gross tumor during IMRT treatment for NPC could lead to ipsilateral parotid glands move into the high-dose region at the first 15 fractions. Which subsequently leads to considerable inaccuracies in dose delivery to ipsilateral parotid glands during IMRT for nasopharyngeal carcinoma. Therefore, measurements of medial edges of parotid gland and GTVnx volume reduction may be a potential helpful strategy for optimizing treatment planning for IMRT and selecting patients who could benefit significantly from adaptive strategies.

## References

[1] Sriuranpong V, Mutirangura A, Gillespie JW, Patel V, Amornphimoltham P, Molinolo AA, Kerekhanjanarong V, Supanakorn S, Supiyaphun P, Rangdaeng S (2004). Global gene expression profile of nasopharyngeal carcinoma by laser capture microdissection and complementary DNA microarrays. Clin Cancer Res.

[2] Al-Sarraf M, LeBlanc M, Giri PG, Fu KK, Cooper J, Vuong T, Forastiere AA, Adams G, Sakr WA, Schuller DE (1998). Chemoradiotherapy versus radiotherapy in patients with advanced nasopharyngeal cancer: phase III randomized Intergroup study 0099. J Clin Oncol.

[3] Lee N, Harris J, Garden AS, Straube W, Glisson B, Xia P, Bosch W, Morrison WH, Quivey J, Thorstad W (2009). Intensity-modulated radiation therapy with or without chemotherapy for nasopharyngeal carcinoma: radiation therapy oncology group phase II trial 0225. J Clin Oncol.

[4] Nishi T, Nishimura Y, Shibata T, Tamura M, Nishigaito N, Okumura M (2013). Volume and dosimetric changes and initial clinical experience of a two-step adaptive intensity modulated radiation therapy (IMRT) scheme for head and neck cancer. Radiother Oncol.

[5] Berwouts D, Olteanu LA, Duprez F, Vercauteren T, De Gersem W, De Neve W, Van de Wiele C, Madani I (2013). Three-phase adaptive dose-painting-by-numbers for head-and-neck cancer: initial results of the phase I clinical trial. Radiother Oncol.

[6] Bhide SA, Davies M, Burke K, McNair HA, Hansen V, Barbachano Y, El-Hariry IA, Newbold K, Harrington KJ, Nutting CM (2010). Weekly volume and dosimetric changes during chemoradiotherapy with intensity-modulated radiation therapy for head and neck cancer: a prospective observational study. Int J Radiat Oncol Biol Phys.

[7] Lee C, Langen KM, Lu W, Haimerl J, Schnarr E, Ruchala KJ, Olivera GH, Meeks SL, Kupelian PA, Shellenberger TD (2008). Evaluation of geometric changes of parotid glands during head and neck cancer radiotherapy using daily MVCT and automatic deformable registration. Radiother Oncol.

[8] Hansen EK, Bucci MK, Quivey JM, Weinberg V, Xia P (2006). Repeat CT imaging and replanning during the course of IMRT for head-and-neck cancer. Int J Radiat Oncol Biol Phys.

[9] Barker JJ, Garden AS, Ang KK, O’Daniel JC, Wang H, Court LE, Morrison WH, Rosenthal DI, Chao KS, Tucker SL (2004). Quantification of volumetric and geometric changes occurring during fractionated radiotherapy for head-and-neck cancer using an integrated CT/linear accelerator system. Int J Radiat Oncol Biol Phys.

[10] Yang H, Hu W, Ding W, Shan G, Wang W, Yu C, Wang B, Shao M, Wang J, Yang W (2012). Changes of the transverse diameter and volume and dosimetry before the 25th fraction during the course of intensity-modulated radiation therapy (IMRT) for patients with nasopharyngeal carcinoma. Med Dosim.

[11] Wang W, Yang H, Hu W, Shan G, Ding W, Yu C, Wang B, Wang X, Xu Q (2010). Clinical study of the necessity of replanning before the 25th fraction during the course of intensity-modulated radiotherapy for patients with nasopharyngeal carcinoma. Int J Radiat Oncol Biol Phys.

[12] Yang H, Tu Y, Wang W, Hu W, Ding W, Yu C, Zhou C (2013). A comparison of anatomical and dosimetric variations in the first 15 fractions, and between fractions 16 and 25, of intensity-modulated radiotherapy for nasopharyngeal carcinoma. J Appl Clin Med Phys.

[13] Nutting CM, Morden JP, Harrington KJ, Urbano TG, Bhide SA, Clark C, Miles EA, Miah AB, Newbold K, Tanay M (2011). Parotid-sparing intensity modulated versus conventional radiotherapy in head and neck cancer (PARSPORT): a phase 3 multicentre randomised controlled trial. Lancet Oncol.

[14] Kam MK, Leung SF, Zee B, Chau RM, Suen JJ, Mo F, Lai M, Ho R, Cheung KY, Yu BK (2007). Prospective randomized study of intensity-modulated radiotherapy on salivary gland function in early-stage nasopharyngeal carcinoma patients. J Clin Oncol.

[15] Ho KF, Marchant T, Moore C, Webster G, Rowbottom C, Penington H, Lee L, Yap B, Sykes A, Slevin N (2012). Monitoring dosimetric impact of weight loss with kilovoltage (kV) cone beam CT (CBCT) during parotid-sparing IMRT and concurrent chemotherapy. Int J Radiat Oncol Biol Phys.

[16] van de Water TA, Bijl HP, Westerlaan HE, Langendijk JA (2009). Delineation guidelines for organs at risk involved in radiation-induced salivary dysfunction and xerostomia. Radiother Oncol.

[17] Yang H, Hu W, Wang W, Chen P, Ding W, Luo W (2013). Replanning during intensity modulated radiation therapy improved quality of life in patients with nasopharyngeal carcinoma. Int J Radiat Oncol Biol Phys.

[18] Zhao L, Wan Q, Zhou Y, Deng X, Xie C, Wu S (2011). The role of replanning in fractionated intensity modulated radiotherapy for nasopharyngeal carcinoma. Radiother Oncol.

